# Post-wildfire water quality and aquatic ecosystem response in the U.S. Pacific Northwest: science and monitoring gaps

**DOI:** 10.1088/3033-4942/ae36cb

**Published:** 2026-02-10

**Authors:** Sara Wall, Jana E Compton, Ashley A Coble, Beth M Haley, Jiajia Lin, Allison Myers-Pigg, Justin Reale, Katie Wampler, Allison Swartz, Kevan Moffett, Kevin D Bladon, Kurt Carpenter, Heejun Chang, Junjie Chen, David Donahue, Chris S Eckley, Amanda K Hohner, Peter M Kiffney, Lorrayne Miralha, Peter Regier, Joshua Seeds, Mark River

**Affiliations:** 1 Pacific Northwest Research Station, Olympia, WA 98512, United States of America; 2 Pacific Ecological Systems Division, Corvallis, OR 97333, United States of America; 3 National Council for Air and Stream Improvement, Inc. (NCASI), Corvallis, OR 97330, United States of America; 4 U.S. Environmental Protection Agency, Pacific Ecological Systems Division, Newport, OR 97365, United States of America; 5 Oregon Department of Environmental Quality, Water Quality Division, Portland, OR 97232, United States of America; 6 Marine and Coastal Research Laboratory, Pacific Northwest National Laboratory, Sequim, WA 98382, United States of America; 7 U.S. Geological Survey, Oregon Water Science Center, Portland, OR 97204, United States of America; 8 Forest Ecosystems and Society, Oregon State University, Corvallis, OR 97331, United States of America; 9 School of the Environment, Washington State University, Vancouver, WA 98686, United States of America; 10 School of Earth, Environment, and Society, Portland State University, Portland, OR 97201, United States of America; 11 Oregon Department of Water Resources, Salem, OR 97301, United States of America; 12 Drinking Water Protection, Eugene Water & Electric Board, Eugene, OR 97402, United States of America; 13 U.S. Environmental Protection Agency, Region-10, Seattle, WA 98101, United States of America; 14 Department of Civil Engineering, Montana State University, Bozeman, MT 59717, United States of America; 15 NOAA Fisheries, Northwest Fisheries Science Center, Fish Ecology Division, Seattle, WA 98112, United States of America; 16 Department of Food, Agricultural and Biological Engineering, The Ohio State University, Columbus, OH 43210, United States of America; 17 Weyerhaeuser Company, Eugene, OR 97405, United States of America

**Keywords:** wildfire, water, nutrients, carbon, metals, hydrology, gap analysis

## Abstract

An increase in the occurrence of large, high severity wildfires in the western Pacific Northwest (PNW), USA, has created an urgent need for science to better inform forest management and policy decisions to maintain source water quality in the region. The western PNW faces similar challenges to other regions with shifting wildfire regimes and large population centers reliant on surface water from forested catchments. However, the uniquely wet and highly seasonal climate of the western PNW suggests that findings from other, more frequently burned regions may not be directly applicable. To identify science, monitoring, and management gaps and opportunities in the western PNW, this review was collaboratively undertaken by academics, non-government and industry representatives, and local, state, and federal government entities who have been working together since the 2020 Labor Day fires in Oregon. Focusing on Oregon and Washington, we found that monitoring networks for continuous water quantity and quality cover much of the state with greater representation in western U.S. ecoregions, but few studies have analyzed and published these data to capture and communicate the post-wildfire response. Approximately half of the streamgages in Oregon and Washington record major water quality parameters, and hundreds of sites in the area have discrete sampling for a wide range of water quality constituents. Still, numerous gaps exist in understanding the short- and long-term impacts of wildfire on hydrology, water chemistry, including pH and dissolved oxygen, mobilization of metals, aquatic ecosystems, and downstream drinking water treatment. Collective action to further collect, analyze, interpret, and publish the key data could help improve our understanding of post-wildfire water quality impacts in this and other increasingly wildfire-affected regions.

## 1. Introduction

**Figure 1.**
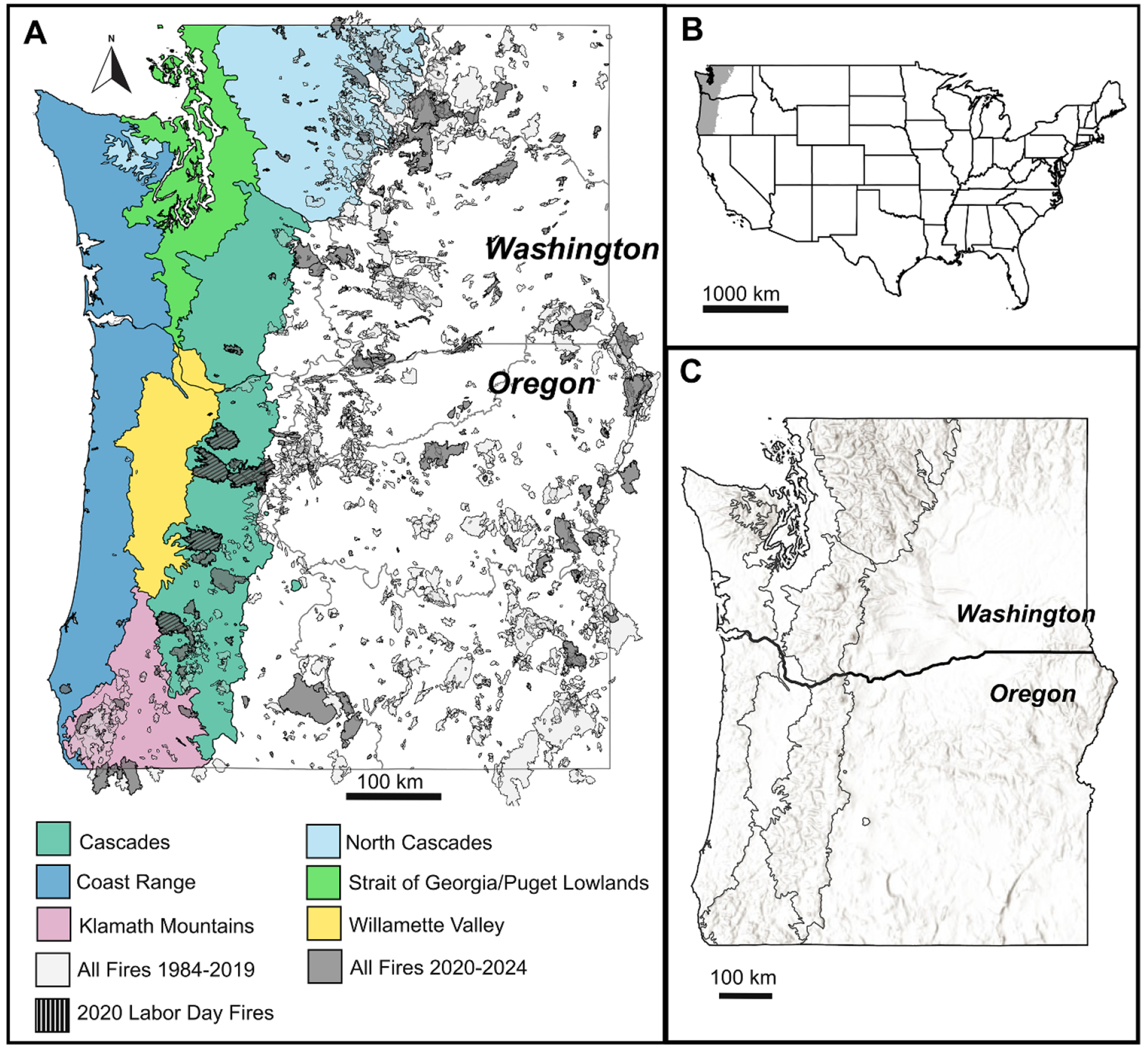
(A) Ecoregions of the western Pacific Northwest in Washington and Oregon, USA (Level III Ecoregions; Omernik 1987), and fire perimeters greater than 405 ha (equivalent to 1000 acres) (1984–2022; Eidenshink et al 2007). The map shows more large fires during and since 2020 in the highlighted ecoregions than in the preceding 36 years. The 2020 Labor Day fires are shown by the striped pattern. (B) Map of the continental United States with the study region shaded in gray. (C) Hillshade map of Washington and Oregon (Esri 2025). Ecoregions of the western Pacific Northwest are outlined in gray.

Global wildfire activity has intensified over the past two decades, with many countries experiencing a significant rise in large, stand-replacing wildfires. This trend is particularly evident in forested and temperate regions, where wildfire potential has increased (Liu et al 2010, Bowman et al 2020, Zhang et al 2023). In the western U.S., there has been a substantial escalation in the frequency and severity of high-intensity fires in the 21st century (Halofsky et al 2020). Previously, forests on the slopes of the Coast Range and western Cascade Range in the U.S. Pacific Northwest (PNW) in the last few centuries have had particularly low fire occurrence due to the temperate rainforest climate and infrequent fire return interval (Reilly et al 2017, 2022), fire suppression activities (Marlon et al 2012), and relative rarity of lightning-caused ignitions (Kalashnikov et al 2023). Increased droughts and warming temperatures are now leading to longer wildfire seasons (Westerling et al 2006, Halofsky et al 2020, Reilly et al 2022). Of note, one powerful storm during extreme weather conditions (high winds, dry fuels, and low humidity) in September 2020 caused numerous wildfires to ignite throughout western Oregon and into northern California, many of which grew into megafires and caused large amounts of destruction (Reilly et al 2022). The 2020 Labor Day megafires (>10 000 ha burned; Linley et al 2025) (figure 1) were spurred by strong dry easterly winds, low humidity, and intense drought (Reilly et al 2022). Six wildfires burned a total of 334 549 hectares (ha) in a few weeks, amounting to ~83% of the total area burned in the western PNW over the previous 30 years combined (Reilly et al 2022). Since 2020, the region has continued to experience large, high severity wildfires, with those between 2021 and 2024 burning another cumulative 1 845 158 ha. These wildfires have caused substantial damage in Washington and Oregon, including loss of life, burned structures, and widespread smoke impacts (Governor’s Wildfire Economic Recovery Council 2021, Filonchyk et al 2022).

Wildfires have the potential to lead to long-term watershed impacts, including water quality concerns for ambient waters and drinking water, flooding, and erosion (Bladon et al 2014, Paul et al 2022). Shifting wildfire regimes are increasing risk to PNW forests (Gao et al 2021) that support recreation, fisheries, energy production, agriculture, cultural values, and drinking water for millions of people (Brown et al 2008, Brandt et al 2014). Several reviews have examined wildfire effects on water resources at regional to global scales (Smith et al 2011, Reale et al 2015, Rhoades et al 2019, Emmerton et al 2020, Barron et al 2022, De Palma-Dow et al 2022, Paul et al 2022, Chen and Chang 2023) and have highlighted impacts on aquatic ecosystems and fish populations (Bixby et al 2015, Gomez Isaza et al 2022, Roon et al 2025), drinking water (Hohner et al 2019, Schulze and Fischer 2021, Pennino et al 2022), wildland-urban interfaces (Magliozzi et al 2024), and the role of land management on post-wildfire impacts (Dobre et al 2022, Beyene et al 2023). Many of these studies report long-term watershed impacts after wildfire, including drinking water quality degradation and increased risk of flooding and erosion (Bladon et al 2014, Paul et al 2022).

The impacts of wildfire on water quality in the western PNW remains understudied relative to the present need (Elliott et al 2024). The timeline of modern intensive settlement, drinking water infrastructure, and western U.S. water quality science in the region is short (~160 years) compared to the timeline for natural fire rotation in the seasonal temperate rainforests (200–1000 years; Donato et al 2020). With concurrent fire suppression practices, there has been little opportunity during the last century to study fire-water relations using contemporary water quality methods. The uniquely wet and highly seasonal climate of the western PNW also suggests that findings from other, more frequently burned areas may not be directly applicable to this region, highlighting the need for region-specific research. This gap in scientific knowledge also parallels a critical shortfall in addressing the needs of forest, water, and public safety managers in response to wildfire in the western PNW.

We reviewed current literature and existing datasets to characterize how fresh waters in the western PNW have responded to wildfire. We focused on water quality, including what we refer to as the ‘Big 5’ water quality parameters (temperature, turbidity, specific conductance, dissolved oxygen (DO), and pH), as well as metals (including mercury), nutrients and organic matter, and aquatic ecology. We also included studies of wildfire impacts on streamflow, post-wildfire forest management implications, and drinking water treatment and quality. We emphasized research conducted between 2015 and 2025, particularly focusing on studies since the 2020 Labor Day fires in western Oregon. This review aims to summarize the current state of knowledge, to identify persisting knowledge gaps, and to outline priorities and cross-cutting issues that impact multiple constituents for future research on wildfires and water quality in the western PNW.

## 2. Review approach

### 2.1. Study area

The western PNW is a biophysically diverse region that spans wide elevational and climatic gradients. Our study focused on the forested areas west of the Cascade Range crest in Oregon and Washington, USA, including the north and western Cascade Range, and Coast Range (figure 1). In this region, rainfall can exceed 2000 mm yr^−1^, forest biomass density is among the highest on the planet, and precipitation is highly seasonal (wet winters, dry summers), with wet springs leading to abundant fuels in the dry summer period (Buma et al 2019, Reilly et al 2022). Forests in this region are particularly at risk from climate change because they span the rain/snow transition zone, making snowpack vulnerable to warming winter temperatures and earlier spring snowmelt (Westerling et al 2006, Buma et al 2019). Less than 10% of precipitation falls during summer months, resulting in particularly dry summers (DellaSala et al 2011, Reilly et al 2017). Within the cool and wet season, atmospheric river events contribute at least 45% of total precipitation, bringing extreme rainfall to low elevations, snowfall to high elevations, and occasional rain-on-snow events near the snowline (Rutz et al 2014, Collow et al 2020). Given the strong seasonality of precipitation and runoff governing element fluxes in the western PNW (Compton et al 2020), and the key role of infrequent, large events on transport in general (Raymond et al 2016), changes in climate, wildfire regime, and post-wildfire hydrology will have important implications for water quality and aquatic ecosystems in the region. Meanwhile, the major economic and population centers in the region, in the Willamette Valley and Puget Lowlands, are supported almost entirely by drinking water systems originating in the forested watersheds of the western Cascade Range (Robichaud and Padowski 2024).

Historical fire regimes in the western PNW have ranged from frequent, low severity fire regimes in the drier parts of the region such as the Willamette Valley and Klamath Mountains to infrequent, stand-replacing wildfire regimes in cooler, wetter areas such as the Coast Range and western Cascade Range (figure 1; Reilly et al 2022). In these wetter areas, fire return intervals historically ranged from 150 to over 400 years, with some evidence suggesting fire return intervals of up to 1000 years (Donato et al 2020). While the 2020 Labor Day fires were much larger than other fires in this region in recent history, they were consistent in size and severity with the largest historical fires in this area over the last century (Donato et al 2020, Reilly et al 2022). In particular, western Oregon experienced multiple large, high severity fires in the watersheds of the Clackamas (Riverside fire), Santiam (Beachie Creek and Lionshead fires), McKenzie (Holiday Farm fire), North Umpqua (Archie Creek fire), Rogue (Almeda and South Obenchain fires) and coastal Salmon (Echo Mountain Complex fire) Rivers. Higher temperatures, decreasing summer precipitation, and increasing drought in the PNW are projected to increase the frequency, extent, and severity of wildfire (Halofsky et al 2020).

### 2.2. Monitoring network assessments

Understanding the magnitude, timing, and duration of post-wildfire impacts on water quality and freshwater ecosystems often depends on the collection of pre- and post-wildfire data (Murphy et al 2023). Globally, water quality monitoring networks are generally less comprehensive in spatial and temporal coverage than streamflow monitoring networks (Zhi et al 2024). This limitation is critical in the context of wildfires, where the unpredictable nature of wildfire events make the spatial and temporal resolution of continuous or long-term discrete monitoring essential for assessing wildfire impacts on water quality, both after wildfire and in preparation for future fires and management scenarios (Murphy et al 2023, Tunby et al 2023). In this review, we assessed the existing monitoring network within the states of Oregon and Washington to identify opportunities for assembling, processing, and analyzing these data streams.

#### 2.2.1. Continuous water quality monitoring

**Figure 2.**
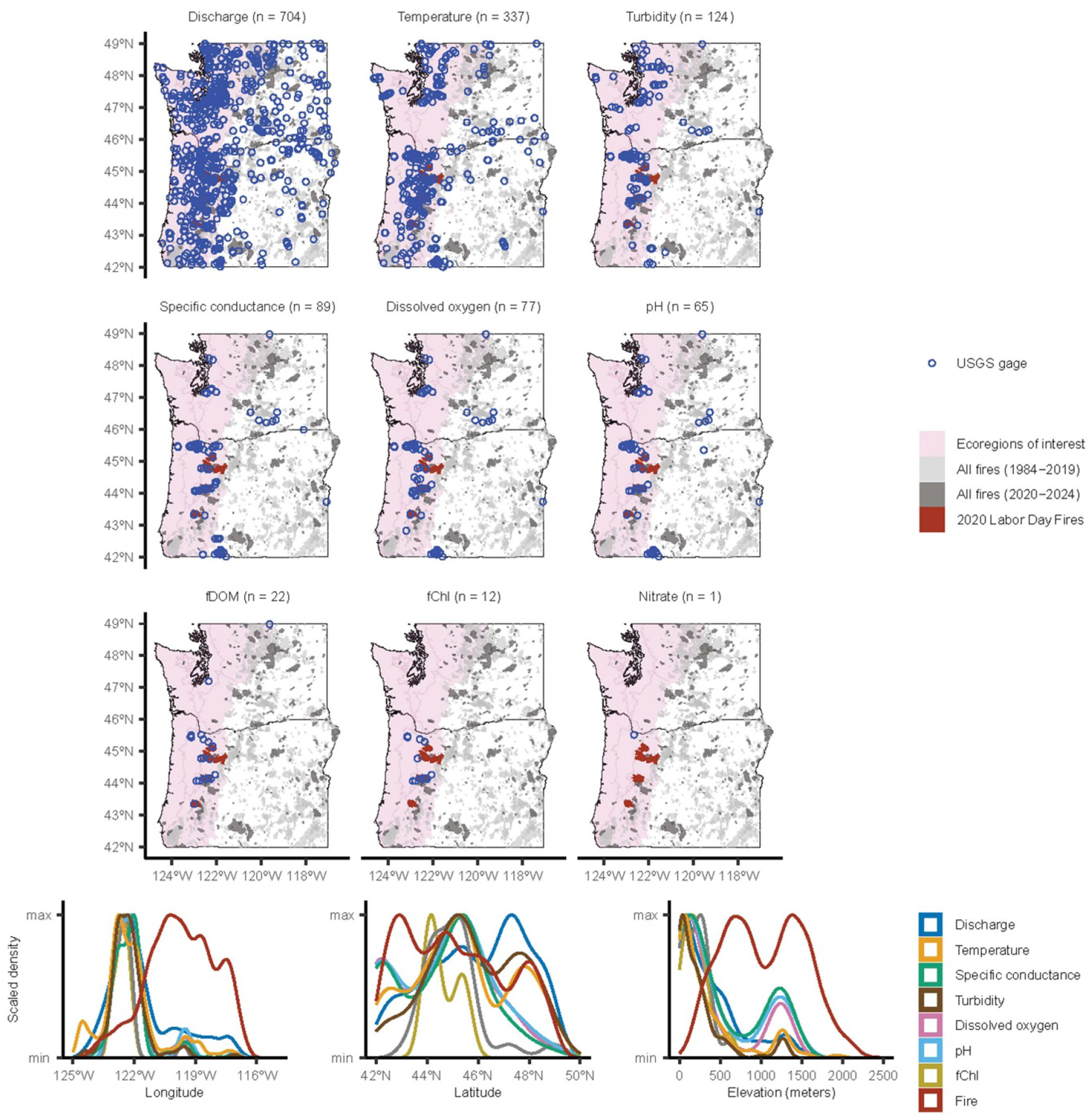
(Top) U.S. EPA Level 3 Ecoregions within the study area within Oregon and Washington (Omernik 1987); USGS continuous discharge, temperature, turbidity, specific conductance, dissolved oxygen, pH, dissolved organic matter fluorescence (fDOM), chlorophyll fluorescence (fChl), and nitrate monitoring stations with records for at least 1 year between 1984–2025 from NWIS (USGS 2025a), and fire perimeters (>405 ha, 1984–2024). (Bottom) Scaled kernel density plots of latitude, longitude, and elevation distributions for each water quality parameter of interest and of the centroids of all fires. In map titles, *n* = number of USGS stations. Nitrate was excluded from density plots as *n* = 1.

To assess continuous monitoring within the study area, we mapped U.S. Geological Survey (USGS) streamgages that primarily measure stream discharge, temperature, turbidity, specific conductance, DO, pH, and less frequently measure dissolved organic matter fluorescence, chlorophyll fluorescence, or dissolved nitrate, at sub-hourly intervals (figure 2) using the dataRetrieval package (De Cicco et al 2025) in R (R Core Team 2024). The *whatNWISdata* function was used to import available parameters, period of record, count, coordinates, and elevation for each streamgage from the USGS National Water Information System (NWIS; USGS 2025a) web services (https://waterservices.usgs.gov/docs/site-service/). We included streamgages that had at least one year of continuous data collected between 1984 and 2025, to align temporally with the Monitoring Trends in Burn Severity dataset (MTBS; Eidenshink et al 2007). The centroid of each MTBS fire perimeter was determined using the terra package (Hijmans 2025). The elevatr package (Hollister 2023) was used to obtain the elevation of the fire perimeter centroid using the USGS Elevation Point Query Service (USGS 2025b). Though our study focused on the western PNW (figure 2), we analyzed streamgage and fire locations across all of Washington and Oregon to compare between subregions and assess broader gaps. While other continuous monitoring networks exist across this region, we chose to focus on the USGS network because it is publicly available and spans the entirety of our study region.

#### 2.2.2. Discrete water quality monitoring

**Figure 3.**
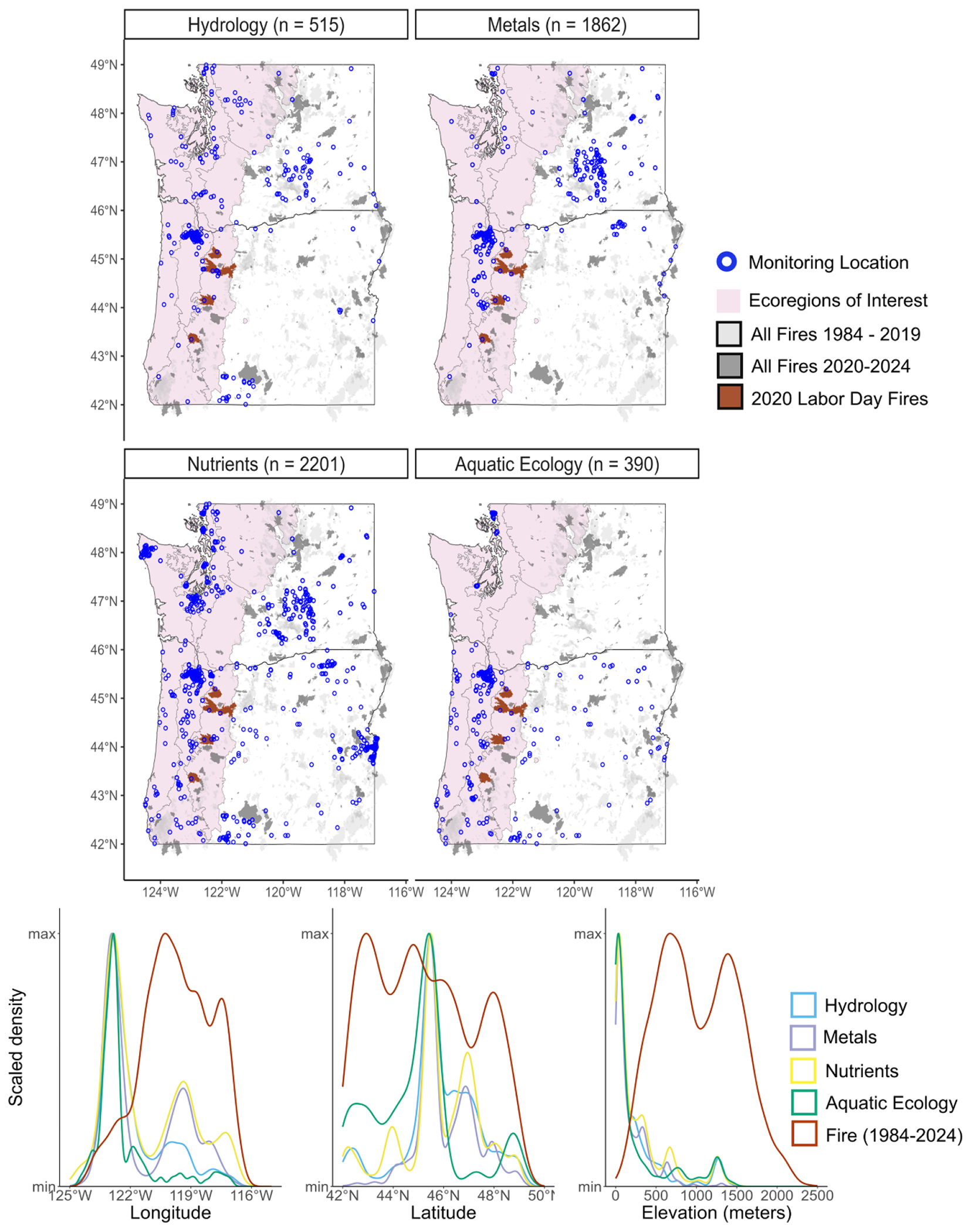
(Top) Study area (pink), discrete water quality monitoring locations from Water Quality Portal (blue dots; Water Quality Portal 2021), and fire perimeters (>405 ha within Washington and Oregon 1984–2024; gray and red). In map titles, *n* = number of monitoring sites. (Bottom) Scaled kernel density plots of latitude, longitude, and elevation for each water quality category of interest and of the centroids of all fires.

Monitoring of water quality constituents that do not have adequate *in situ* real-time continuous methods brings additional challenges, including the expense of field crews for discrete sample collection, laboratory infrastructure for sample preparation, analysis, quality assurance, and data assembling and sharing in a timely manner. Because of the difficulty in compiling discrete data from multiple sources with differing data availability, compatibility, methods, and documentation, we chose to analyze discrete monitoring data collected and compiled only by the National Water Quality Monitoring Council through their Water Quality Portal (WQP; Water Quality Portal 2021). The WQP combines publicly available water quality data from the USGS NWIS and the U.S. Environmental Protection Agency Water Quality Exchange Data Warehouse (Water Quality Portal 2021). Data within the WQP is submitted by states, Tribes, watershed groups, other federal agencies, volunteer groups, and universities and is publicly accessible. We included sites that were sampled greater than 40 times between 1984–2025 and grouped the parameters into the following categories, aligned with the subsequent structure of this paper: hydrology, metals, nutrients, and aquatic ecology (figure 3; SI table 1). We excluded ‘Big 5’ parameters because they are most often and more thoroughly measured using continuous methods and are highlighted in figure 2.

## 3. Results and discussion

### 3.1. Hydrologic connections to water quality

#### 3.1.1. Wildfire effects on hillslope hydrology and streamflow

Wildfire can lead to changes in watershed rainfall-runoff responses and streamflow, typically exhibiting increased peak, low, and annual flows, water yields, and surface runoff (Moody et al 2013, Hallema et al 2018, Pirani and Coulibaly 2025). Short-term streamflow dynamics after the 2020 Labor Day fires in Oregon have shown patterns similar to other regions (Collar et al 2022), with greater burned area (>20%) driving reduced evapotranspiration and increased runoff (Long and Chang 2022, Kang et al 2024). There are many effects on in-stream flow and water quality (Partington et al 2022), with shifts in hydrology typically linked to decreased evapotranspiration (ET) from vegetation loss (Ma et al 2020, Collar et al 2021); reduced infiltration from wildfire-induced soil water repellency, pore clogging, and structural degradation of soils (DeBano 2000, Larsen et al 2009, Ebel and Moody 2017); and shifts in snow dynamics (Goeking and Tarboton 2020). Shifts in rain/snow precipitation proportions and snow dynamics are particularly salient for the wet forests of the western PNW and Chile (Buma et al 2019), and likely also the few other seasonal temperate rainforests of the world. These shifts in hydrology may alter the quantities and timing of water quality constituents delivered to receiving waters (Paul et al 2022), with varying periods of hydrologic recovery to pre-wildfire ranges reported across studies (Robichaud 2000, Cerdà and Doerr 2005, Wine and Cadol 2016).

Modeling and empirical studies indicate wildfire severity metrics and watershed and post-wildfire climate characteristics (aridity, annual precipitation, soil type, vegetation type, geologic province) were important predictors of annual and peak flow hydrological response to wildfire in the western PNW (Wampler et al 2023). In the first two post-wildfire years, runoff ratio increased, and evaporative index (ET/Precipitation) decreased in burned watersheds, with these changes attributed to reductions in ET (Kang et al 2024). Burned forests of the western Cascade Range have been shown to experience greater snow accumulation, ablation rates and earlier snow disappearance for at least several years after wildfire (Gleason and Nolin 2016, Uecker et al 2020). Although hydrological and geomorphological responses in the first two years following the 2020 Labor Day fires were muted (Busby and Wilcox 2024), this was attributed to below average precipitation, absence of extreme precipitation events, and precipitation intensities remaining below thresholds known to trigger debris flows in the first two years following the fires (Busby and Wilcox 2024). Peak flows in the first two years post-wildfire remained below the 2 yr recurrence interval in Holiday Farm and Archie Creek study areas and generally water year (WY) 2021 was drier than WY 2022 but neither experienced rainfall intensities that would trigger landslides or channel reorganization events (Busby and Wilcox 2024). Variation in post-wildfire precipitation amounts and timing can moderate streamflow and erosion.

#### 3.1.2. Gaps and opportunities

Within the western PNW, an extensive network of streamgages can rapidly capture post-wildfire streamflow changes, which simultaneously reveals a significantly greater real-time monitoring for water quantity *versus* quality (figure 2; Murphy et al 2023, Zhi et al 2024). To address important questions of scale and seasonality, nested streamgages with paired water quantity and quality data collection are needed. Postwildfire studies often require utilization of existing infrastructure with readily available pre- and post-event data. However, streamgages in the western PNW overrepresent large river systems at lower elevations and underrepresent smaller streams and hillslope processes more completely altered by wildfire, as depicted by elevation in figure 2 and consistent with broader evaluations of USGS streamgages (e.g. Deweber et al 2014). As part of this much-needed hillslope and headwaters research, weather data should connect local rainfall intensities to downstream risks of landslides, debris flows, and other catastrophic water quality-disrupting events (Collow et al 2020, Collar et al 2022, Selander et al 2025), with associated water quantity and quality gages informed by such linkage.

Overall, a more holistic and mechanistic understanding of how hydrological responses to wildfires may differ in the western PNW compared to other regions is needed to be able to interpret and, eventually, connect to the short- and long-term responses of solutes and aquatic ecosystems in the region’s waters. While there is recent progress investigating short-term (2 year) hydrological responses to wildfire, there remains a need to investigate longer-term hydrological, geomorphological, and solute dynamics that evolve over time (Long and Chang 2022). More empirical data are needed in this region to accurately parameterize post-wildfire vegetation, soil, and hillslope conditions in hydrological models to better support runoff, erosion, and water quality prediction (Saxe et al 2018, Pacheco and Fernandes 2021, Ebel et al 2023).

### 3.2. The ‘Big 5’ water quality parameters: temperature, specific conductance, turbidity, DO and pH

#### 3.2.1. Wildfire effects on temperature

Water temperature is a major driver in the growth, tolerance, and distribution of aquatic organisms, biogeochemical reaction rates, and ecosystem processes (Leach et al 2023). Although recent global reviews reported that water temperature predominantly increases post-wildfire (Paul et al 2022, Chen and Chang 2023), thermal effects and recovery to pre-wildfire ranges are complex. Initially, decreases in temperature within and outside of the burn perimeter can occur during wildfire from smoke and reduced solar input as observed in California (David et al 2018) and Oregon streams (Sanders et al 2022), which is also influenced by pre-wildfire riparian canopy type and removal (Isaak et al 2010, Dahm et al 2015, Rosenberger et al 2015), altered channel morphology (Dunham et al 2007), and salvage logging (Chen and Chang 2023). Following wildfire, stream temperatures often increase, but not always (Dahm et al 2015). Thermal sensitivities of streams post-wildfire can vary with elevation, topographic shading, groundwater inputs, and riparian shade (Chen and Cheng 2023).

Few studies have investigated post-wildfire stream temperature responses in the western US. Specific to the PNW, Beyene and Leibowitz (2024) observed a 0.3–1 °C increase in daily summer water temperatures at 31 burned sites within the PNW. Within our western PNW study region, Sanders et al (2022) documented brief and mixed thermal effects on the day the 2020 Labor Day fires burned the riparian area adjacent to a specific water temperature sensor, along with a cooling effect from smoke across all sites. In contrast, during the summer immediately following the Labor Day fires, stream temperatures exceeded 16 °C for ~80% of the time and 20 °C for ~30%, representing a 6–7 °C increase from prewildfire conditions (Warren et al 2022). Collectively, the effects of the Labor Day fires on stream temperature were greater in more severely burned watersheds across Riverside, Beachie Creek, and Holiday Farm fires (Coble et al 2023). Within the Clackamas River Basin, temperatures in the Riverside fire were explained by upstream burned area, elevation, and the percent of upstream volcanic geology of the Cascade Range (Krochta and Chang 2024). Recent studies have examined the potential impacts of climate change on stream temperatures in burned watersheds in this region, projecting greater warming in these areas by the mid to late 21st century (Chen and Chang 2023, Krochta et al 2025).

#### 3.2.2. Wildfire effects on turbidity, suspended sediment, and suspended solids

Wildfires can alter sediment transport in streams and rivers (Shakesby and Doerr 2006, Sankey et al 2017), often increasing suspended sediment concentrations and loads (Malmon et al 2003, Reale et al 2015, Curtis et al 2025). The magnitude and duration of post-wildfire sediment pulses are influenced by regional factors such as climate, topography, burn severity, and the timing and intensity of subsequent storms (Cannon et al 2010, Ryan et al 2024). Burned watersheds typically exhibit sharper, more frequent turbidity spikes following precipitation, particularly in areas with high burn severity, slow vegetation recovery, or both (Hall et al 2022). Turbidity response is often most pronounced immediately following wildfire, with sediment concentrations regressing over subsequent years (Desilets et al 2007). Channels are often dominant sediment sources, with post-wildfire yields from channelized flows exceeding those from hillslopes (Moody and Martin 2009). Ephemeral streams also demonstrate distinct sediment transport behaviors after wildfire, including peak concentrations at the leading edge of flood waves (Malmon et al 2003).

Burned watersheds within the western PNW have shown greater turbidity during storm events compared to unburned basins, at least over the short-term (Chen and Chang 2025). Similarly, turbidity-estimated suspended sediment concentrations were greater post-wildfire than pre-wildfire in two study watersheds within the Lionshead and Beachie Creek fire boundaries (Clark et al 2025). A meta-analysis across the western U.S. from before the 2020 Labor Day fires indicated that post-wildfire turbidity and sediment fluxes in the PNW may be more episodic and less sustained than in arid regions, although the analysis included only a few eastern Coast Range sites from the western PNW at the time (Moody and Martin 2009). The porous, organic-rich soils and steep western PNW forested catchments may provide greater hillslope sediment storage capacity and less tendency for spatially extensive post-wildfire soil water repellency, rill, and gully formation than in drylands (Jackson and Roering 2009); however, understanding post-wildfire sediment loading and debris flow processes across largely unmonitored and extensive rural PNW forest areas is still a rapidly expanding area of research (Burns et al 2025).

#### 3.2.3. Wildfire effects on specific conductance

Specific conductance is a measure of the dissolved ionic content of waters and provides information on mixing of water masses, mobilization of soil salts and ash, and other wildfire-relevant aquatic processes. Wildfires influence conductivity primarily through changes in hydrology, including runoff ratios and flowpaths, and through mobilization of dissolved and solid materials, primarily soils and ash.

Of the studies conducted in the U.S. and reviewed by Paul et al (2022), conductivity generally increased post-wildfire (11 of 16 studies), with one study reporting decreased conductivity, and 4 of 16 studies reporting no change. Wildfire impacts on specific conductance are generally short-lived, persisting less than 5 years on average, and may lag due to the timing and amount of precipitation post-wildfire (Reale et al 2015). The literature reporting wildfire impacts on specific conductance in the PNW is limited, with none of the studies covered in the review by Paul et al (2022) overlapping with our study region (figure 1). However, mountainous areas in the PNW are prone to debris flows after wildfire (as reviewed by Wondzell and King 2003), which transport ions, carbon, and nutrients into river networks. For example, a post-wildfire runoff-initiated debris flow was observed after the Milli fire in the western Oregon Cascade Range (Wall et al 2020), and pre- and post-wildfire debris flow hazards were also assessed after the Labor Day fires (Selander et al 2025). The surface of the burned landscape may also play a large role, with a layer of ash above a burned soil surface, for example, contributing to higher specific conductance of runoff, at least for eastern Cascade Range soils (Ahmed and Hassan 2023).

#### 3.2.4. Wildfire effects on DO and pH

DO and pH are important indicators of stream metabolism, revealing the metabolic regime of photosynthetic organisms and respiration of aquatic ecosystems (Bernhardt et al 2018, Jankowski et al 2021). DO is also influenced by physical processes such as turbulence, temperature, and organic and inorganic materials in the water (Hall et al 2016). DO availability and pH both influence the mobility and toxicity of pollutants, affect drinking water treatment and infrastructure, and are non-enforceable secondary drinking water contaminants (US EPA 2015). These parameters and associated biogeochemical processes are impacted by wildfires (Dahm et al 2015, Reale et al 2015, Emmerton et al 2020) with effects sometimes extending 100+ kilometers downstream of burn perimeters as observed in New Mexico (Nichols et al 2024) and throughout the western U.S. (Ball et al 2021).

Interpretations of continuous DO and pH data following wildfire within the study region are limited. Sanders et al (2022) collected DO data prior to, during, and following the 2020 Holiday Farm fire in 8 streams and observed a daily minimum DO as low as 17% of saturation on the day of the fire. The site-averaged daily minimum DO on the day of the fire remained above 7.0 mg L^−1^, which is above the State of Oregon regulatory threshold (7 d absolute minimum of 6 mg L^−1^ ; Oregon Administrative Code 340-041-0016). The decline was attributed to smoke effects and recovered over several days. A series of rain events within ~2 weeks after the same fire again resulted in episodic declines in DO, but DO still remained above 7.5 mg L^−1^ and 80% of saturation. Coble et al (2023) collected DO data at 24 sites 8–11 months after the 2020 Holiday Farm, Beachie Creek, and Riverside fires, but the DO data were reported only by site as a 7 d minimum. The USGS also measured continuous pH and DO at 7 sites within a burn perimeter prior to, during, and following the 2020 Labor Day fires (USGS 2021). Western PNW studies of *in situ* freshwater pH are even more limited, but ex situ tests of Cascade Range burned soils and ash suggested that ash may promote higher runoff pH, at least in the short term of the first few post-wildfire rainfall events (Ahmed and Hassan 2023).

#### 3.2.5. Gaps and opportunities

As evident from the limited literature within the study region, there are opportunities to further evaluate the effects of wildfire on the ‘Big 5.’ For example, the proliferation of multi-parameter water quality sensor networks over the last several decades provide the opportunity to further evaluate wildfire effects on the ‘Big 5.’ Within the study region, the continuous water quality data streams maintained by the USGS offer an opportunity to address these knowledge gaps (figure 2). Additional resources are needed to delineate wildfire-impacted water quality streamgages, identify monitoring bias (such as ecozone, longitude, and elevation; figure 2), harmonize datasets, and evaluate meteorological and landscape conditions prior to and following wildfire in the western PNW. Further deployment of rapid response continuous monitoring streamgages (Tunby et al 2023) could substantially improve our understanding of wildfire impacts within the study area but requires the continued support to keep streamgages operational in dynamic post-wildfire hydrologic conditions (Ball et al 2021, Murphy et al 2023, Nichols et al 2024). In addition, analysis of the existing USGS data, in particular DO, pH, and conductivity data, could address some of the important gaps in response of these fundamental constituents.

### 3.3. Aquatic metals

#### 3.3.1. Wildfire effects on metals

Wildfires can increase the transport of metals and metalloids from burned landscapes to streams in forests worldwide (Neary et al 2005, White et al 2006), including in the western U.S. (Abraham et al 2017, Rust et al 2018, 2019). After a wildfire, metals bound in plant or soil organic matter become concentrated in ash (Cerrato et al 2016, Sanchez-Garcia et al 2023, Marcotte et al 2025). Metals such as iron, manganese, arsenic, chromium, aluminum, barium and lead then accumulate in fine sediments and are eroded into surface waters during heavy rainfall (Smith et al 2011, Bladon et al 2014, Burton et al 2016). Wildfire may increase the toxicity of metals such as chromium (Lopez et al 2023, 2024) and alter organic matter to influence metal redox reactions and availability (Lopez et al 2024). Concentrations of copper, lead, iron and zinc increased after wildfire in western U.S. forested watersheds (Stein et al 2012, Pinedo-Gonzalez et al 2017), while others found little change in concentrations of arsenic, copper, iron, manganese, mercury, nickel, and silver (Mast et al 2016). Concentrations of metals of concern such as copper, iron, lead and zinc have increased in drinking water sources after fires (Paul et al 2022); for example, arsenic was particularly elevated in public water supplies from groundwater after fires in the western U.S. (Pennino et al 2022). Wildfires can also mobilize metals from incineration at other land uses within the watershed, such as from mining waste, automobiles, or housing materials (Murphy et al 2020, Magliozzi et al 2024).

Except for mercury (refer to next section), we did not identify published studies documenting post-wildfire changes in freshwater metal concentrations in the western PNW. In general, metals are less well-studied than other water quality parameters such as nutrients, turbidity, and organic carbon (Paul et al 2022). While drinking water providers and other efforts may have measured stream or river aquatic metal concentrations since the 2020 Labor Day fires in the western PNW; these results have not been published but may be published in the future.

#### 3.3.2. Wildfire effects on mercury

The impact of wildfires on mercury (Hg) mobilization differs from that of other metals/metalloids for several reasons. Mercury is the only metal that can exist as a low-temperature gas (as elemental Hg^0^), which allows for a long atmospheric residence time and potential for deposition in areas remote from emission sources (Schroeder and Munthe 1998, Strode et al 2008). Most human and ecological risks are from its organic form, methylmercury (MeHg), which is formed microbially, has increased toxicity, and biomagnifies in aquatic food webs (Hsu-Kim et al 2013, Eagles-Smith et al 2018). Ecosystem changes that alter microbial communities, including wildfire, can therefore increase (or decrease) MeHg production and accumulation in downstream ecosystems (Hsu-Kim et al 2018, Jeong et al 2024). Wildfires release Hg stored in surface soils and vegetation to the atmosphere (Webster et al 2016, McLagan et al 2021) and promote erosion of Hg-containing soils into streams. Enhanced leaching of nutrients and labile organic matter after wildfire can increase microbial methylation and associated aquatic bioaccumulation (Kelly et al 2006, Li et al 2022). As a result, post-wildfire landscapes often have lower soil Hg concentrations (Mitchell et al 2012, Homann et al 2015, Webster et al 2016), while post-wildfire streams have higher Hg and MeHg concentrations (Burke et al 2010, Stein et al 2012, Campos et al 2015, Jensen et al 2017) compared to pre-wildfire conditions.

The western Cascade Range (figure 1) is an area of elevated atmospheric mercury deposition and accumulation in soils and vegetation due to the high amount of rainfall and locally effective sequestration of deposited Hg in organic material (Obrist et al 2004, Domagalski et al 2016). Although wildfire may remove some Hg from the soil and vegetation to the atmosphere, post-wildfire precipitation and runoff may continue to increase Hg levels in streams due to enhanced erosion and/or decreased sequestration of deposited Hg. For example, Hg concentrations in the soil O-horizon decreased by over 88% following a wildfire in Oregon, with most of the loss attributed to releases to the atmosphere (Homann et al 2015). However, streams in western Oregon showed 89 and 178% higher concentrations of particulate Hg and MeHg, respectively, in recently burned catchments compared to unburned catchments, with higher concentrations associated with greater burn severity and greater bioaccumulation in some macroinvertebrates (Baldwin et al 2024).

#### 3.3.3. Gaps and opportunities

Additional studies could help increase understanding of post-wildfire temporal trends in metal mobilization, stream metal concentrations, forms, and bioavailability. Additional studies could also help increase understanding of hydrological and chemical drivers, transformations, and interactions of wildfire-liberated aqueous metals with microbial communities, source materials, and organic matter. Given the known complexity of wildfire effects on mercury cycling, and the potential unknown complexities of other less studied metals’ fates after wildfire, additional stream water quality monitoring would be needed to assess burned landscapes’ long-term tendencies to sequester or supply Hg and other metals to the fresh waters of the western PNW. Such monitoring could help evaluate concurrent post-wildfire mobilization of other nutrients from burned catchments, such as sulfate, which may stimulate microbial communities responsible for metals’ transformations.

### 3.4. Aquatic nutrients and organic matter

Wildfires often increase the mobilization of nutrients, ions, and organic matter from watersheds to aqueous environments, sometimes by many orders of magnitude, even to levels that challenge drinking water treatment (Paul et al 2022). However, observed responses can be specific to constituents, watersheds, and burn characteristics (Raoelison et al 2023, Cavaiani et al 2024). Most post-wildfire studies and meta-analyses focus on concentrations of dissolved constituents rather than particulate-bound materials. Generally, these studies have found that nutrient and organic matter concentrations increase within a year post-wildfire and typically can remain elevated for about 5 years following the wildfire based on reviews from the western U.S. (Rust et al 2018), North America (Cavaiani et al 2024), and globally (Raoelison et al 2023).

Here, we focus primarily on major nutrients—nitrogen (N) and phosphorus (P), and organic matter—that serve as fuel for food webs, contribute to downstream algal blooms (Rhea et al 2021), and affect drinking water systems (Hohner et al 2019). We also review current knowledge of major ions that can influence water quality following wildfire.

#### 3.4.1. Wildfire effects on nitrogen and phosphorus

Large increases in N and P concentrations are common after wildfire, with 77% and 65% of studies reporting post-wildfire increases, respectively (Paul et al 2022). For example, a study in Colorado, USA found that nitrate and total dissolved nitrogen (TDN) increased by 500%–1600% after wildfire (Rhoades et al 2019), and nitrogen species (ammonia, nitrate, total nitrogen, organic nitrogen) also tended to increase following wildfires in other regions (Rust et al 2018, Uzun et al 2020). A study in northern California suggested that the impacts of wildfire on nitrogen species could last <2 years (Uzun et al 2020), while an analysis of 159 wildfires in the western U.S., found ammonia, nitrate and organic nitrogen to be correlated with the percent of area burned at moderate and high severity for five years post-wildfire (Rust et al 2019).

Studies on in-stream nitrogen in the western PNW have been limited to the first three years following wildfire where increases in nitrate, dissolved organic nitrogen, and total nitrogen have been observed (Roebuck et al 2022, Bush et al 2024, McCredie et al 2025). Such increases in N are often positively correlated with burn severity (Roebuck et al 2022, Coble et al 2023). This is consistent with previous work in other systems. However, in the first post-wildfire precipitation event following the 2020 Holiday Farm wildfire, TDN was observed to decrease with higher burn severity (Roebuck et al 2022). In the first summer following the 2020 Labor Day fires, variations in stream water nitrogen concentrations were explained by an interaction between burn severity and stand age, with older stands exhibiting lower nitrate and TDN concentrations post-wildfire, while ammonia concentrations did not vary as a function of wildfire severity (Coble et al 2023).

In the western PNW, significant increases in phosphate were observed during the first flushing period post-wildfire, compared to the pre-wildfire period (Bush et al 2024), consistent with previous work in other ecosystems. These increases remained relatively steady throughout the entire season (Bush et al 2024). Despite this, total phosphorus (TP) and phosphate concentrations across western Oregon were not related to burn severity during the first summer post-wildfire in one study (Coble et al 2023), but another study that spanned the western U.S. found that TP was correlated with the percentage of area burned at moderate to high severity for years 3–10 post-wildfire (Rust et al 2019). Across the western U.S., total dissolved P only significantly increased in 18.2% of 18 wildfire sites (Rust et al 2018), while a study from central Idaho found that orthophosphate did not differ among burned and unburned sites four years post-wildfire (Harris et al 2015).

#### 3.4.2. Wildfire effects on organic carbon and organic matter composition

Increases in organic carbon post-wildfire has been observed in ~50% of studies in the western U.S. (Paul et al 2022). While shifts in dissolved organic carbon (DOC) are often smaller than those observed for other nutrients and ions (Paul et al 2022), work in Idaho (Minshall et al 2001, Harris et al 2015) and California (Uzun et al 2020, Richardson et al 2024) has typically observed increases in DOC following wildfires. Notably, only minimal shifts in DOC concentrations have been observed in western PNW watersheds, contrary to many studies in other regions. Large storms are expected to drive large (>5 mg l^−1^) but short-term increases in DOC concentrations as has been observed in Utah and Colorado, USA and Alberta, Canada (Emelko et al 2011, Murphy et al 2015, Crandall et al 2021). Concurrently, with increases in DOC, we would expect wildfire to increase aqueous carbon aromaticity due to inputs of aromatic pyrogenic organic matter (Wagner et al 2015). Organic matter composition is often characterized by absorbance and fluorescence measurements and indices. Increases in SUVA254 (specific ultra-violet absorbance at 254 nm divided by DOC concentration) and fluorescence index are most often observed after wildfires as found in California (Uzun et al 2020) and Colorado (Hohner et al 2016, Fischer et al 2023), indicating an increase in more terrestrial or lower molecular weight, aromatic-like organic matter.

During the first flushing period following the 2020 Labor Day fires, a non-significant decrease in DOC was observed (Roebuck et al 2022, Bush et al 2024). Similarly, in the third year post-wildfire, DOC concentrations decreased with increased burn severity. However, this effect was only significant during the fall flushing period (Wampler et al 2024). Conversely, during the dry summer period of the first year post-wildfire, a small (<1.5 mg l^−1^) increase in DOC (and DON) was observed, covarying with increasing burn severity (Coble et al 2023). The differences between DOC observations in other regions and the western PNW suggest that DOC responses are unique to the western PNW region, with regional and local characteristics such as precipitation patterns, vegetation, and hydrology strongly controlling the post-wildfire patterns (Wampler et al 2024).

Studies following the 2020 Labor Day fires suggest that DOM composition is related to burn severity (Roebuck et al 2022, Wampler et al 2025), with an increase in aromatic, polycondensed compounds and nitrogen-containing compounds with increased burn severity (Roebuck et al 2022). However, this trend was not observed using SUVA254 during baseflows (Coble et al 2023). Additionally, post-wildfire DOM compositional shifts also appeared to be seasonally dependent with shifts primarily observed during the dry summer and fall flushing period (Wampler et al 2025).

#### 3.4.3. Wildfire effects on major dissolved ions

Anion and cation concentrations and fluxes are often elevated after wildfire, including for Cl^−^, SO_4_^2−^, Ca^2+^, Mg^2+^, and K^+^ (Mast and Clow 2008, Santos et al 2019, Bush et al 2024). In a review of wildfire in the western U.S., aqueous fluxes of nutrients, anions, cations, and metals all increased after burning, although our study region was not included in their analysis (Rust et al 2018). For SO_4_^2−^ and Ca^2+^, concentrations were lower but fluxes were higher after wildfire due to increased discharge, whereas both concentrations and fluxes increased for Mg^2+^, Na^+^, and K^+^ in most watersheds evaluated (Rust et al 2018). After wildfire in Montana SO_4_^2−^ and Cl^−^ concentrations were elevated for two years (Mast and Clow 2008). Bedrock lithology may determine whether cations increase by affecting chemical weathering rates (Santos et al 2019) and may also indicate sources such as surface, subsurface or deep groundwater (Knapp and Musolff 2022). In Colorado, ash deposition and smoke contributed to increased K^+^ concentrations prior to post-wildfire precipitation events (Rhoades et al 2025).

In the H.J. Andrews Experimental Forest of western Oregon, NO_3_^−^, PO_4_^3−^ and SO_4_^2−^ concentrations in streams increased significantly in two watersheds with low burn severity, while K^+^ increased in only one of the watersheds; Mg^2+^, Ca^2+^, and Cl^−^ did not change significantly (Bush et al 2024). Prior reviews of major ion response to wildfire did not have representation from western Oregon or western Washington (e.g. Rust et al 2018), and more empirical research could help understand whether ion responses behave similarly in this region after wildfire and to identify mechanisms of such changes.

#### 3.4.4. Gaps and opportunities

Despite substantial research on nutrient responses to wildfire in forested watersheds, there remain several questions about responses in systems with long wildfire return intervals, such as the western PNW. In this region, N, P, and C are the best studied nutrients and organic matter, while others—such as major ions and various macro- and micronutrients—remain comparatively understudied. Nutrient data are often collected through grab sampling, which can result in significant spatiotemporal gaps, particularly during storm events and periods of high streamflow and sediment transport. However, the post-wildfire deployment of high-frequency optical sensors for nitrate and DOM provides opportunities for continuous and spatially comprehensive monitoring (Murphy et al 2023). Understanding the impacts of wildfire and predicting recovery timelines to pre-wildfire ranges remain challenging given the complex landscapes, which include repeated and sometimes prescribed burns, and a lack of pre-wildfire monitoring data. Numerical modeling approaches informed by satellite-derived landscape attributes (e.g. burn severity, vegetation dynamics) may be one solution to disentangle wildfire impacts from shifts in N, P, and C due to changing climate and land use.

### 3.5. Aquatic ecosystem ecology

Wildfire affects aquatic ecosystems both directly and indirectly, through altering riparian conditions, changing habitat structures, and shifting watershed inputs (Gresswell 1999, Erdozain et al 2024). Alterations to physical and chemical conditions can be pronounced but are also dynamic through time and space depending on burn severity, riparian and upland recovery, and post-wildfire weather events; this results in complex responses across trophic levels (Roon et al 2025). Severe wildfires can change soil properties, kill overstory riparian vegetation, increase solar radiation, alter large wood recruitment, increase discharge (section 1), nutrient (section 4), and sediment delivery (section 2), and alter DOM character (section 4), which in turn affect invertebrate, fish, and amphibian populations.

#### 3.5.1. Wildfire effects on aquatic ecosystem function

Wildfires that alter the balance of nutrient and energy demand via changes in molar stoichiometry or changes in DOM character will likely affect in-stream processing of nutrients and DOM. Nutrient spiraling studies have shown nutrient uptake efficiency decreases with greater background dissolved inorganic nitrogen (DIN) concentrations in the absence of wildfire (Peterson et al 2001, Dodds et al 2002, Ensign and Doyle 2006) as well as after wildfires (Rodríguez-Cardona et al 2020). To our knowledge, changes in in-stream nutrient demand following wildfire have not been explored in the PNW, which has substantially lower concentrations of DOM than studies from Arctic systems that are often referenced (Diemer et al 2015, Rodríguez-Cardona et al 2020). Yet, observed reductions in DOC and increases in N in the western PNW (refer to section 3.4) have likely reduced the DOC:TN or DOC:DIN stoichiometric ratios, potentially affecting DOM composition (Wampler et al 2025). Basal resources are critical for fueling aquatic food webs, and wildfire may enhance gross primary production if mortality of overstory riparian trees increases light availability; however, wildfire may also enhance ecosystem respiration if more labile organic matter is delivered to streams. Labile sources of C, including portions of the pyrogenic C continuum (Myers-Pigg et al 2015), may stimulate ecosystem metabolism including processing of more recalcitrant DOM via the priming effect (Bianchi 2011, Masiello and Louchouarn 2013). In western Oregon, elevated light availability, stream temperature, and potentially nutrient delivery to streams resulted in greater autotrophic production in post-wildfire conditions (Swartz and Warren 2022) consistent with prior observations from other regions (Malison and Baxter 2010, Silins et al 2014), but ecosystem metabolism responses have not been studied in the western PNW.

#### 3.5.2. Wildfire effects on aquatic biota

Periphyton, macroinvertebrate and fish responses after wildfire are largely affected by conditions during flood events (low DO, high turbidity, substrate movement) and through recovery post-wildfire (Dunham et al 2007, Tuckett and Koetsier 2016). After the 2020 Labor Day fires, macroinvertebrate densities increased in more severely burned watersheds in the first summer after wildfire but experienced decreased diversity and reductions in scrapers, intolerant, and sensitive taxa (Coble et al 2023), which is consistent with studies from other regions (Mellon et al 2008, Verkaik et al 2015). Fish and amphibian responses to wildfire are mixed in the literature (Hossack and Pilliod 2011, Rieman et al 2012), ranging from extensive mortalities to increased densities (Howell 2006, Dunham et al 2007, Sestrich et al 2011), likely in part confounded by additional habitat changes post-wildfire (flooding, drought, pest outbreaks etc). Fish and amphibians in western Oregon after the 2020 Labor Day fires persisted in equal or greater densities compared to pre-wildfire conditions (Swartz and Warren 2022, Warren et al 2022). In the first three years post-wildfire, greater fish and total vertebrate densities were observed in systems experiencing greater wildfire severity (Swartz et al 2025). In many studies where large impacts on aquatic ecosystems have been observed, wildfires were followed by high-flow events causing flooding and channelization (Burton 2005, Leonard et al 2017, Gomez Isaza et al 2022). Following the 2020 Labor Day fires, burned areas did not experience high intensity rain events or flooding, but were subjected to both pre- and post-wildfire drought (Six et al 2025). Understanding how the unique climate of moist coniferous forests may interact with wildfire relative to findings from drier regions could aid our understanding of potential resilience of these systems to future wildfire events.

#### 3.5.3. Gaps and opportunities

For the western PNW, major gaps in our understanding of aquatic ecosystem ecology responses include effects of wildfire alone, intersection with major post-wildfire hydrologic events, and influence of compounding disturbances. In the western PNW, rain is typically received in moderate intensity that may limit adverse effects of multiple compounding events, although pre- and post-wildfire drought is a compounding concern regionally. Ecosystem function (nutrient spiraling, metabolism) response to wildfire is also largely unknown in the PNW, but understanding how these processes respond to wildfire is an important missing link to identify mechanisms to explain observed biological responses to wildfire. Further, ecosystem metabolism responses during wildfires likely vary depending on post-wildfire debris flows (Tuckett and Koetsier 2016) and riparian vegetation recovery (Davis 2015, Finkler et al 2025), geology and stream size (Davis 2015), and the degree of loose organic matter (Robinson et al 2005) and likely vary over time during the months and years following wildfires, but these factors have not been explored in the western PNW. Proliferation of multi-parameter water quality sensor networks over the last several decades provide the opportunity to further evaluate wildfire effects on ecological processes such as stream metabolism. For example, compilation and analysis of high-frequency datasets with pre- and post-wildfire records from USGS, National Ecological Observatory Network (www.neonscience.org/), and StreamPULSE (https://data.streampulse.org/) provide a robust opportunity to examine post-wildfire changes in aquatic ecosystem function (Appling et al 2018), which may be further strengthened if coupled with remote sensing of downstream impacts (e.g. algae blooms; Olson et al 2023). In addition, we know little about the effects of wildfire, including extent, timing, and severity on fish growth, movement, and survival. Finally, harmful algal blooms (HABs) have potential aquatic ecosystem and drinking water implications (Brooks et al 2016, Clark et al 2017), but more work is needed to strengthen understanding of post-wildfire trends in HABs in the western PNW (Olson et al 2023, Carpenter et al 2025).

### 3.6. Post-wildfire management action effects on water quality

#### 3.6.1. Post-wildfire forest management strategies

Post-wildfire harvest of burned trees (salvage logging) is a frequently used management tool in the western PNW to recover some economic value from killed or damaged trees, while also seeking to reduce risk of subsequent disturbances such as pest outbreaks and re-burns (Leverkus et al 2018). Salvage logged areas are replanted with tree seedlings as required by forest practice regulations, such as Oregon Forest Practice Administrative Rules and the Forest Practices Act (Oregon Administrative Rules 629, Divisions 600 through 680). Following the 2020 Labor Day fires, salvage logging followed by reforestation efforts occurred broadly across private, state, and federal lands typically within 1–2 years post-wildfire. Prior to salvage logging, road maintenance was prioritized, and culverts and road crossings were replaced. Other strategies implemented in the aftermath of the Labor Day fires included aerial seeding of unsalvaged upland or riparian areas and in some cases underplanting of unsalvaged areas to promote reforestation. In un-salvaged and unmanaged areas, where no active planting or seeding occurred, natural regeneration of the existing seed bank will determine vegetation recovery trajectories. Drinking water utilities also can rapidly implement forest land source water restoration strategies, such as installing sediment fences or restoring vegetation as implemented by Eugene Water & Electric Board after the Labor Day fires, to limit adverse effects on drinking water intakes in the aftermath of wildfires (Toth et al 2024).

#### 3.6.2. Hydrologic, stream sediment, and stream temperature response to post-wildfire forest management strategies

Due to limited information on the effects of salvage logging within the western PNW, we summarize research from other regions. For a variety of reasons (e.g. soil alterations), wildfires typically increase surface runoff. Long-term hydrologic responses in Washington exhibited hydrologic recovery to pre-wildfire ranges 35–41 years after wildfire in two salvage logged and seeded catchments, while in the burned but unmanaged catchment (not salvaged or seeded), annual flow and runoff ratios remained elevated 35–41 years later (Niemeyer et al 2020). This response was attributed to delayed vegetation recovery in the absence of active management. Conversely, in Alberta, Canada, mean annual discharge 8 years after wildfire was similar in reference, burned, and burned + salvaged catchments, but the range of flows differed among these post-wildfire management strategies with the greatest variability observed in burned and salvaged catchments, followed by burned catchments, and the least variation in unburned reference catchments (Martens et al 2019).

Sediment is among the most significant water pollutants associated with forest management (U.S. Environmental Protection Agency 2005). Soil erosion by surface runoff can be significantly elevated after wildfires when compared to undisturbed forests (Moody and Martin 2009). The practices associated with post-wildfire salvage logging can further increase surface runoff, soil erosion, and mass wasting, and result in more effective terrestrial sediment transport networks (Silins et al 2009). Using heavy equipment, such as feller-bunchers and skidders, during salvage logging can lead to compacted soil and decreased infiltration capacity, therefore enhancing runoff generation and erosion rates (Wagenbrenner et al 2015, Prats et al 2021); however, slash treatment greatly reduced erosion and sediment yield (Prats et al 2021). Wildfires tend to enhance hillslope-water connectivity (Olsen et al 2021), leading to an increased capacity of surface water transferring sediments to downstream locations. Constructing linear features, such as roads and skid trails, during salvage logging further accelerates soil erosion and the delivery of sediment to streams by expanding the connectivity network (Wagenbrenner et al 2023) and providing additional source material from quarries and re-rocking of roads (Luce and Black 1999). This hydrologic alteration forced by road networks can be severe following wildfire (Sosa-Pérez and MacDonald 2017, Wagenbrenner et al 2023). A study in southern Oregon found that salvage logged sites had 3–4 times greater amounts of mean ground erosion and substantial decreases in ground cover (5%–20%) compared to 70%–80% in non-salvaged, unmanaged areas (Slesak et al 2015). Yet, the likelihood and intensity of disturbance-induced sediment transport following wildfire and post-wildfire salvage logging are dependent on soil burn severity and near-term rainfall intensity (Wagenbrenner et al 2023).

There are relatively few studies of salvage logging effects on stream temperature, although research suggests that removing residual shade increases stream temperatures immediately after wildfire. Post-wildfire studies that included sites with salvage logging in the first year after the 2020 Labor Day fires found elevated stream temperatures in the Holiday Farm fire (Swartz and Warren 2022), Archie Creek fire (Warren et al 2022) and throughout Riverside, Beachie Creek, and Holiday Farm fires (Coble et al 2023), but these responses were concurrent with active logging leading to difficulty in isolating the effects of wildfire from salvage. In Alberta, Canada, daily average and maximum temperatures in burned catchments were 0.8 °C and 1.0 °C higher than unburned catchments, respectively (Wagner et al 2014). Daily average and maximum temperatures were 2.1 °C and 3.1 °C higher in burned and salvaged catchments, respectively, compared to unburned controls in this same study, showing additional temperature increases over those caused by wildfire alone (Wagner et al 2014). While stream temperatures were significantly elevated in three western Oregon studies, fish populations persisted in some of the most severely burned catchments that included both salvaged and unsalvaged catchments, but the mechanisms remain unclear (Warren et al 2022, Swartz et al 2025). Longer-term monitoring may aid in understanding the trajectory of temperature and fish recovery to pre-wildfire ranges in severely burned and salvaged watersheds.

#### 3.6.3. Gaps and opportunities

Watersheds harvested after wildfires under confounding effects of both wildfire and logging may be at greater risk of both short- and long-term degradation of aquatic systems and water quality (Emelko et al 2011, Silins et al 2014). It is challenging to distinguish the influence of post-wildfire timber harvest on water quality from the impact of wildfire, in part due to limited availability of pre-wildfire data for comparison, and because wildfire and subsequent post-wildfire salvage disturbances are distributed non-randomly across the landscapes in which they occur. Some observational studies have shown that salvage logging and its associated actions can have long-lasting effects on forest ecosystems including aquatic conditions (Karr et al 2004, Leverkus et al 2018, Nemens et al 2019, Wagenbrenner et al 2023). Multi-catchment analysis reveals that variability of watershed or stream response to forest practices may depend on catchment physiography and lithology (Bywater-Reyes et al 2018) and local weather patterns (Slesak et al 2015). Site-specific assessment, pre-logging planning, and best management practices during and after logging are critical for the sustainability of post-wildfire forest management (Leverkus et al 2021).

Numerous papers have theorized the effects of salvage logging on aquatic ecosystems (Karr et al 2004, Reeves et al 2006), but little empirical data exist, in part due to inability to set up a robust experimental design following such major disturbances. Data collection both prior to and following salvage logging is often logistically challenging due to safety concerns, funding constraints, and restricted land access, all of which can delay the timely establishment of monitoring for burned and managed areas. After a wildfire, there is often limited time between when infrastructure (roads, bridges, culverts) is replaced and barriers (downed trees) are removed to allow safe access, but prior to the initiation of salvage logging (e.g. James and Krumland 2018). Typically, salvage logging occurs immediately after an event, and it is difficult to design a study to encompass pre-wildfire data, pre-salvage data, and post-salvage data relative to suitable control locations to elucidate the role of salvage versus wildfire.

While the effects of salvage logging on sediment are relatively well understood at hillslope plot scales, much less is known about instream responses, such as changes in large wood, temperature, substrate, and aquatic food webs. Knowledge gaps include both immediate and long-term effects on aquatic ecosystems, and there remains a need for information on active versus passive management of riparian areas post-wildfire (e.g. Reeves et al 2006). Long-term monitoring of existing observational studies to study delayed and cumulative ecological responses over time may aid in elucidating differing trajectories of severely burned salvaged versus unsalvaged watersheds or riparian areas (e.g. Niemeyer et al 2020). The magnitude of land area affected by concurrent megafires in western Oregon in 2020 also complicated efforts to adequately design experimental studies. An established network of collaboration among researchers, agencies, land managers, landowners, and other stakeholders prior to fires may better enable flexibility to rapidly deploy an experimental design that incorporates salvage treatments after wildfire. For example, a before-after-control-impact study can aid in teasing apart climate change effects from wildfire and salvage effects. An experimental design that utilizes a controlled burn with salvage and unsalvaged treatments may improve our ability to separate these effects, although controlled burns may not adequately mimic wildfire conditions.

### 3.7. Drinking water

#### 3.7.1. Wildfire effects on drinking water systems

Wildfire impacts on drinking water systems are highly variable and complex, with multiple factors influencing the relative risk, exposure and resiliency each system may experience. Two broad pathways through which wildfires can impact drinking water quality include source water contamination and damaged infrastructure. Wildfires located in areas with drinking water sources can produce contaminants that challenge treatment performance (Hohner et al 2019). Wildfires in forested source watersheds can increase runoff (section 1) and delivery of sediment, metals, organic matter, and nutrients (sections 2–4; Hohner et al 2019, Paul et al 2022) to drinking water intakes. Large fluctuations in source water quality driven by post-wildfire precipitation events can introduce challenges for treatment operations and long-term planning for utilities. High concentrations of sediment can disrupt treatment processes and accumulate in reservoirs (Emelko et al 2011, Hohner et al 2016). Increased concentrations of metals in source water can introduce issues of taste, odor, and toxicity in finished water (Bladon et al 2014, Rust et al 2018). Post-wildfire changes in the quantity and composition of source water DOM can form disinfection byproducts (DBPs; Chow et al 2019, Hohner et al 2019), chemicals formed during treatment when DOM reacts with chemical disinfectants used to address waterborne pathogens. Increases in nutrients, light, and water temperature can lead to HABs and the release of toxins that threaten drinking water quality (Klose et al 2015, Paul et al 2022). Additional chemical contaminants that can enter drinking water sources after wildfire can originate from burnt infrastructure (Wang et al 2022), burnt legacy mining sites (Murphy et al 2020), or potentially from the application of wildland firefighting chemicals (Schammel et al 2024).

The second mechanism impacting post-wildfire drinking water quality consists of direct impacts of wildfire on drinking water distribution infrastructure through ignition or damage from radiant heat. Above ground facilities (e.g. treatment facilities, pumphouses, valves, storage assets) are at risk during a wildfire, and soil thermal propagation can lead to pipe failure or enhanced chemical leaching from heat-exposed pipes in below ground distribution networks (Mitchell et al 2025). Failure of pipes or other critical infrastructure assets can result in depressurization of the distribution system, infiltration by wildfire smoke, total loss of water supply, and/or temporary interruption to service. Chemical leaching from thermally exposed pipes depends on pipe material and other aspects including wildfire characteristics, depth of pipes, and temperature. Chemical leaching in drinking water has primarily been reported in plastic pipes exposed to extreme heat (Proctor et al 2020, Schulze and Fischer 2021, Solomon et al 2021). Chemicals observed in thermally exposed plastic pipes after wildfires and in laboratory studies include benzene, toluene, ethylene, xylene, and other volatile organic chemicals (VOCs) with negative implications for human health (Mitchell et al 2025). Besides chemical presence in drinking water, VOCs can also permeate and diffuse in plastic pipes, requiring flushing of the pipe network before drinking water safety is restored (Toth et al 2024, Mitchell et al 2025).

#### 3.7.2. Wildfire effects on PNW water systems

Many treatment systems in the PNW rely on surface water sources, including large population centers in the region (e.g. Portland, Salem, and Eugene, OR; Seattle, Tacoma, and Everett, WA; Robichaud and Padowski 2024). Of the drinking water utilities in the PNW that rely on surface water and serve more than 10 000 people, 67% only have access to surface water sources, making these systems more vulnerable to wildfire (Robichaud and Padowski 2024). Further, many sources in the PNW have traditionally had low raw water sediment and DOM requiring minimal treatment (e.g. unfiltered, slow sand filtration; Emelko et al 2011, Davis et al 2021). Systems that do not use conventional filtration in their treatment systems have lower thresholds for source water turbidity and DOM (Davis et al 2021) and are less resilient to large turbidity, and in some instances DOM, fluctuations that may occur after a wildfire. Finally, because of the historically low risk of wildfire in the study area, many drinking water utilities were not designed to treat the short- and long-term impacts of wildfires on source water quality (Padowski 2021, Robichaud and Padowski 2024).

Research into the impact of wildfires on drinking water in the PNW remains extremely limited despite widespread documented concern among water utility operators in the western U.S. (Jones et al 2023) and utilities in the PNW more specifically (Padowski 2021). The 2020 Labor Day fires impacted 54 drinking water systems (Seeds et al 2020) and one million Oregonians (Toth et al 2024), and case studies suggest that the primary challenges for drinking water utilities were damage to infrastructure, VOC contamination in the distribution system, and degraded source water (Toth et al 2024). VOCs in distribution systems have been the primary focus of existing work, with a report by Oregon Health Authority Drinking Water Services (2022) finding that in the 25 drinking water systems tested following the 2020 Labor Day fires, VOCs were present at a detectable concentration in 20 systems. Despite this finding, only six systems measured concentrations over the maximum contaminant level (MCL; US EPA 2025), with benzene being the most common compound above the MCL. A case study from Detroit, Oregon, suggests that aggressive flushing of water lines shortly after wildfire can decrease VOCs in the lines and prevent adsorption in the pipes (Toth et al 2024).

Elevated sediment concentrations post-wildfire (section 2) can present significant concern for drinking water systems. Specifically, Detroit, Oregon, was forced to switch to other treatment methods post-wildfire due to high particulate levels in source water streams (Toth et al 2024). While upgrading and expanding treatment processes can address these challenges, the cost to implement infrastructure can be prohibitively expensive, especially for small drinking water systems. An alternative mitigation strategy is preventing post-wildfire erosion and debris flows (section 3.6).

Overall, lessons learned from three Oregon drinking water systems impacted by the Labor Day fires (Toth et al 2024) highlight the need for rapid responses to mitigate impacts from direct damage to drinking water distribution networks, increased sediment loads, and other source water contaminants. Establishing partnerships and trust with landowners and other stakeholders prior to wildfires supports the ability to respond quickly when needed. Larger public water systems that have established continuous drinking water quality monitoring networks from source to treatment can benefit from real-time characterization of water quality conditions before, during, and after wildfire, providing advanced warning of rapidly changing conditions during and following wildfire. However, monitoring can be prohibitively expensive for smaller systems to establish and maintain (Toth et al 2024). Often, critical upstream water quality monitoring infrastructure is destroyed by wildfire, driving the need to maintain portable, continuously logging, and telemetered water quality monitoring stations that can be quickly deployed upstream of the drinking water diversion.

#### 3.7.3. Gaps and opportunities

The response to Oregon’s 2020 Labor Day fires led to lessons in rapid testing and VOC sampling guidance (Oregon Health Authority 2020, 2022), and short- and long-term responses of drinking water utilities that experienced wildfire impacts to infrastructure and source watersheds (Toth et al 2024). However, many gaps remain on the impacts of wildfires on drinking water in the PNW, some of which are tied to uncertainties about how wildfire impacts source water quality. Developing a comprehensive profile of wildfire-associated contaminants that may occur throughout the drinking water system–from source water to treatment and distribution–would aid future drinking water utility responses by prioritizing and streamlining water quality testing. Of particular importance are contaminants that are not regulated–and thus not already part of drinking water compliance sampling–but have implications for human health, such as nitrogen-based DBPs (Hohner et al 2019) and the numerous VOCs that have been detected in water systems with directly impacted infrastructure (Mitchell et al 2025). Addressing uncertainties about the drivers and response of HABs in western Cascade Range reservoirs used for drinking water are particularly important for public water systems (Carpenter et al 2025). Additional research could help systematically understand the magnitude of costs associated with drinking water treatment during and after wildfire and investments to increase resilience in response to increasing wildfire risk and associated benefits (Wibbenmeyer et al 2023).

### 3.8. Monitoring network coverage and gaps

#### 3.8.1. *Pre-wildfire* in situ *monitoring resources*

Across the categories of data reviewed in this study (aquatic ecology, ‘Big 5’, metals, nutrients, hydrology and other constituents), spatial variability of monitoring locations is high (figures 2 and 3). Out of the 1431 USGS streamgages analyzed in the continuous monitoring analysis, nearly half measured discharge (figure 2). The other half measured at least one of the ‘Big 5’ parameters. Less than 3% of the continuous USGS streamgages in Washington and Oregon measure anything other than hydrology and ‘Big 5’ indicators. Out of the entire USGS continuous monitoring network assessed across Washington and Oregon, 72% of streamgages are located within our study region (figure 2). Out of the 4,968 discrete data collection locations across Washington and Oregon from the WQP, 44% are for nutrients and ~37% are for metals related data collection, which cover all ecoregions in our study region (figure 3).

In addition to the high spatial variability among indicators and water quality categories, there is also a divide in where monitoring occurs and where wildfire is burning (figures 2 and 3). Most monitoring takes place on the west side of the Cascade Range, while the majority of wildfire occurs on the east side of the Cascade Range (figure 1). There is also an elevational divide in both the continuous and discrete monitoring networks, where lower elevations in the study region are more densely monitored, while wildfire tends to burn in higher elevation areas (figures 2 and 3). This spatial mismatch between where most fires have historically occurred and where most of monitoring data are collected is converging because wildfire is increasing in size and severity on the west side of the Cascade Range (Reilly et al 2017, Halofsky et al 2020). Therefore, this abundance of monitoring on the west side of the Cascade Range may provide opportunities to assess wildfire effects on surface water in this region in the future.

#### 3.8.2. Post-wildfire in situ monitoring resources

The current continuous and discrete monitoring networks may capture the rapid post-wildfire data collection that is necessary to detect immediate (1 day to 1 month) responses to wildfire in select catchments, but resources are needed to analyze and publish findings. Shortly after the 2020 Labor Day fires in Oregon, a rapid and informal post-wildfire data collection mobilization developed across agencies and included inter-agency coordination, leveraging different kinds of monitoring, and direct coordination among academic and government scientists and drinking water managers (Wall and Halofsky 2024). Subsequently, several state and federal agencies, along with universities, Tribes, and other organizations, formed the Oregon Post-wildfire Research and Monitoring Collaborative (Oregon Post-wildfire Collaborative). The goals of the Oregon Post-wildfire Collaborative were to foster awareness of participant activities and to coordinate post-wildfire research and monitoring efforts across disciplines and boundaries. Because wildfires and watersheds extend across land ownership and management boundaries, developing connections and collaborations can enhance our ability to effectively and rapidly monitor burned landscapes. Additionally, compiling datasets across projects can be a challenge in addressing watershed- or wildfire-wide assessments of post-wildfire impacts. To address this challenge, the Oregon Post-wildfire Collaborative developed a catalog of research and monitoring efforts occurring after the 2020 Labor Day fires (Wall and Halofsky 2024). This catalog aims to promote collaboration and reduce redundancy in monitoring. Collaboration and the leveraging of available resources across groups and disciplines can increase efficiencies and improve both rapid post-wildfire response and the ability to maintain such monitoring efforts for years post-wildfire.

## 4. Conclusions: opportunities for future science and monitoring

Monitoring locations in the western PNW have relatively good spatial coverage, particularly for continuous water quantity and quality monitoring data (figures 2 and 3). Discrete data that are not continuously collected (such as metals) are not as well monitored. The network is largely ad-hoc, with individual sites and parameters added over time to address various, locally specific issues or studies, and there is a disconnect between monitoring data collection and the broad-scale analysis, interpretation and publication of these data. While a substantial amount of water quality data has been collected by local, state, and federal entities, there is a gap in analysis and communication of these data. The goal of this review was to identify opportunities for assembling, processing, and analyzing these data streams.

Our analysis identified gaps in research, monitoring, and data analysis for hydrology, water chemistry, and aquatic ecosystem response to wildfire (table 1), and for post-wildfire management and drinking water (table 2). The gaps were grouped into the following categories: (1) careful design of research (R), (2) increased frequency and spatial extent of monitoring (M), and (3) analysis of existing data streams (A). Primary science gaps in understanding and management of water quality post-wildfire in the western PNW include identifying the nature of these science needs (R, M, A), opportunities to address these gaps, and cross-cutting issues that apply to and can impact multiple constituents.

### Research gaps:

Research gaps refer to collection of data with a specific design to address the science gaps identified here. Delineating the wildfire-affected sites within a burn perimeter is straightforward, but characterizing downstream impacts of fires is more challenging. Understanding long-term responses is important but is limited by the short time frame since the recent Labor Day fires, necessitating continued monitoring at key sites. More research is needed using multiple parameters, including a need for co-location of hydrology, ‘Big 5’ and aquatic ecosystem responses to identify connections and drivers of post-wildfire responses. Using StreamPULSE data and approaches may allow determination of where these changes are observed downstream (Bernhardt et al 2022). In addition, many of the existing analyses provide information on response to wildfire in general, but a cross-cutting gap is improved insights on the effects of wildfire size, wildfire history, and burn configuration within watersheds on the water quality response.

### Monitoring gaps:

Monitoring data offer context for evaluating the effectiveness of management and restoration efforts. Long-term streamgage records often are found on large rivers instead of small, low-order streams with watersheds that are more likely to burn and at higher intensity. Data from small, high elevation streams are more difficult to obtain even prior to the fires, even though high elevation areas are more likely to burn (figures 2 and 3). Monitoring networks within the study region are most concentrated within the Puget Lowlands and Willamette Valley ecoregions, which are areas with the highest population densities. These ecoregions, however, are at low elevations that have experienced much less wildfire than other ecoregions within the study area, presenting a disconnect between where most monitoring is taking place and where wildfire has occurred (figures 2 and 3). In addition, more effort is needed to prioritize placement of water quality streamgages related to monitoring bias, and co-locating water quality and quantity collection.

### Data processing and analysis gap:

Given the amount of existing data identified in this review, there are substantial opportunities to obtain, clean (e.g. quality assurance, comparability of methods, consistency of units), and analyze these data and address many of the gaps identified, including event-based or spatially informed data integration. In particular, the utilization of such datasets in biogeochemical numerical modeling can significantly improve predictive understanding of wildfire impacts on water quality.

Executing numerical modeling approaches to simulate streamflow and water quality dynamics both before and after wildfire has been feasible for decades; however, the quantity of data necessary to adequately calibrate and validate such models for specific water quality constituents in the geographic areas of concern is often cited as generally lacking (Ebel and Moody 2020, Ebel et al 2023). Therefore, in addition to synthesis of existing data identified in this review, remotely sensed datasets and counterfactual simulations may be vital for disentangling wildfire impacts from watersheds and land use effects (Ebel et al 2023, Wampler et al 2025).

Despite a large amount of existing continuous and discrete data, no publications were found to have examined changes in conductivity, metals, DO, and pH post-wildfire in the region. In addition to data identified through the USGS real-time and the WQP data sets, drinking water providers collect time-intensive, high quality and relevant raw water monitoring data representing an opportunity for others to assist the utilities in assembling, cleaning, and providing these data to others for further analysis, including enhancing numerical model parameterization.

#### Targeted research recommendations:

To aid in elucidating the effects of wildfire, pre- or post-wildfire management, and climate interactions, we recommend targeted approaches that include (1) experimental designs such as before-after-control impact designs of paired watersheds with staggered implementation, (2) experimental laboratory and field studies targeting ash leachates, soil hydrophobicity, and DOM/metals mobilization (e.g. Ebel and Moody 2017, Roebuck et al 2025) (3) regional ecohydrologic modeling that utilizes remote sensing tools such as burn severity, vegetation recovery, and climate regimes to separate wildfire from climate and land use effects, and (4) data integration that uses event-condition analyses (storm versus baseflow), seasonal stratification, and combined continuous sensors with discrete sampling.

#### Cross-cutting issues:

Numerous cross-cutting questions spanning multiple constituents and categories addressed in this paper remain unanswered, and addressing these questions could improve our understanding of post-wildfire changes and inform watershed and drinking water system management. Differences in the overall size and severity of wildfires, confounded with spatial biases in the existing monitoring network, complicate efforts to find appropriate pre-wildfire data for comparison. Others have identified challenges to post-wildfire monitoring that are relevant for the western PNW, including hydrogeomorphic effects on instrumentation and infrastructure (Murphy et al 2023, Nichols et al 2024). The intersection of historical forest management, repeated fires on the same landscape (reburns), climate dynamics, and variations in post-wildfire salvage logging create a complex temporal and spatial context that makes interpreting post-wildfire responses particularly challenging. The historical distribution of large wildfires in this region (Reilly et al 2022) leads to difficulties in finding suitable control or reference sites for comparison. A recent assessment of post-wildfire runoff and erosion as related to debris flow hazards and mechanisms in the PNW has highlighted that there is a need for improvements in post-wildfire runoff and erosion hazard forecasting in the western PNW (Selander et al 2025). They found that the distinct initiation mechanisms for post-wildfire debris flow hazards in the western PNW cannot be informed by more commonly studied events in other ecoregions and climates (Selander et al 2025).

In many ways, the western PNW region may be affected by these or future megafires through downstream transport and atmospheric emissions and deposition, creating substantial challenges in understanding and mitigating the post-wildfire responses. Drawing upon the broad community of academic institutions, non-government organizations, industry, and local, state and federal government entities working together in the Oregon Governor’s collaborative and beyond, additional coordinated efforts in monitoring and analysis could help address the most important gaps identified here.

## Supplementary Material

SupplementalTable1

Supplementary material for this article is available online

## Figures and Tables

**Table 1.** Summary of science and monitoring gaps for water quality post-wildfire in the western Pacific Northwest (PNW), identifying the nature of the science needs (M = monitoring, A = analysis of existing data, R = new research), opportunities to address these gaps, and cross-cutting issues (X) impacting multiple constituents.

Post-wildfire science and monitoring gaps within the western PNW		Nature of Gap	Opportunities to address the gaps	Cross-cutting?
Hydrology	Streamgages and weather stations in suitable locations (low vs. high elevation, within burn perimeter)	M	Deployment of rapid-response weather stations and streamgages in areas with higher wildfire risk at higher elevation and smaller streams	X
	Post-wildfire changes in snowmelt timing	R, M	Install streamgages above downstream reservoir operations	
	Intersection between pre- and post-wildfire forest management and watershed characteristics	R	Connecting post-wildfire vegetation, soil, and hillslope conditions as they influence runoff	X

Big5	Comparison of post-wildfire stream temperature changes before and after 2020 Labor Day fires and influence of watershed characteristics on thermal sensitivities	A, R	Continued research and analysis of existing data over the longer-term	
	No published work on specific conductance changes	A	More analysis of the existing data	
	Need to understand post-wildfire response of turbidity and sediment transport to increasing size and severity of fires	M, A	Continued monitoring and analysis over the longer-term	
	Few studies of DO and pH post-wildfire	A	Compilation and analysis of high-frequency datasets with pre- and post-wildfire record	X

Metals	Currently no published studies of metal mobilization, drivers and cycling other than mercury	R, A	Analysis of existing data, more monitoring, especially in developed and wildland-urban interface areas	
	Short-term mercury response is well studied	M, A	Longer-term studies including low-level mercury and methylmercury analysis	

Aquatic nutrients and organic matter	C, N and P are well-studied; better understanding of short- and long-term response dynamics needed	M, A	Use of high frequency, real-time optical sensors for nitrate and dissolved organic matter, connection to drinking water and ecosystems	X
	Connecting these data to aquatic ecosystem response	R	Co-located research on nutrients and aquatic ecosystems paired with hydrology	X
	Changes in the size and intensity of western PNW fires provides challenges for use of prior data	R	Expand spatial and temporal scales of evaluations of nutrient and organic matter recovery to represent biophysical variation within these large wildfires	X

Aquatic Ecosystems	Understanding of aquatic ecosystem ecology responses to wildfire alone and compounding disturbances	R	Design of experiments, data analysis and modeling efforts to disentangle these responses	X
	Ecosystem function and fish response largely unknown	A, R	Expand research on nutrient spiraling, ecosystem metabolism, food web responses	X

**Table 2.** Summary of science and monitoring gaps for forest management and drinking water quality post-wildfire in the western Pacific Northwest (PNW), identifying the nature of the science needs (M = monitoring, A = analysis of existing data, R = new research), opportunities to address these gaps, and cross-cutting issues (X) impacting multiple constituents.

Post-wildfire science gaps for forest management and drinking water within the western PNW		Nature of Gap	Opportunities to address gaps	Cross-cutting?
Post-wildfire management	Limited research that clearly identifies the effects of post-wildfire salvage logging	R, M	Experimental design that utilizes a controlled burn with salvage and unsalvaged treatments	X
	Limited ability to distinguish wildfire and timber harvest effects, forest management legacies	R, M	Establishing connections with land managers prior to disturbance may facilitate experimental studies post-disturbance	X
	Interactions between watershed characteristics (physiography, geology) and management	R, A	Science to support site-specific assessment, planning and best management practices are needed	
	Difficult to adequately design experimental studies in the landscape that isolate wildfire effects from forest management	R, A	Continued observational studies; more paired watershed studies with experiment designs to distinguish impact from different disturbances	X

Drinking water	Best approaches to mitigate the impacts of current wildfires on drinking water infrastructure	R, A	Science to inform rapid responses to protect drinking water source areas and infrastructure	
	Understanding the cost associated with drinking water treatment and recovery during and after fires: inform investments in resilience to wildfire	R, A	Studies of drinking water treatment costs and actions to promote resilience; particularly supporting smaller systems	
	Analysis to identify key water testing to aid future drinking water utility responses	A	Develop comprehensive profile of wildfire-associated contaminants that may occur throughout the drinking water system	
	Upstream source water monitoring during and just after active fires	M	Rapid deployment of portable, telemetered water quality monitoring infrastructure	
	Understand if post-wildfire harmful algal blooms responses have potential aquatic ecosystem and drinking water implications	R, A	More research and analysis of existing data to strengthen understanding of post-wildfire HAB trends	X

## Data Availability

The data that support the findings of this study are openly available at the following URL/DOI: https://waterservices.usgs.gov/docs/site-service/; https://doi.org/10.5066/P9QRKUVJ. Supplemental table 1 available at https://doi.org/10.1088/3033-4942/ae36cb/data1.
