# Supplementary material for: Post-wildfire water quality and aquatic ecosystem response in the U.S. Pacific Northwest: science and monitoring gaps
Source: Environ Res Water. Author manuscript; Available in PMC 2026 May 5. (PMC13137517; doi:10.1088/3033-4942/ae36cb)
Supplement: SupplementalTable1 [file NIHMS2147926-supplement-SupplementalTable1.docx]

***Supplemental Information:***

***Supplemental Table 1: List of discrete water quality parameters represented in each broad category for Figure 3. The data are from the Water Quality Portal.***

**Post-Wildfire Water Quality and Aquatic Ecosystem Response**

**in the U.S. Pacific Northwest: Science and Monitoring Gaps**

*Sara Wall, Jana E. Compton*, Ashley A. Coble, Beth M. Haley, Jiajia Lin, Allison Myers-Pigg, Justin Reale, Katie Wampler, Allison Swartz, Kevan Moffett, Kevin D. Bladon, Kurt Carpenter, Heejun Chang, Junjie Chen, David Donahue, Chris S. Eckley, Amanda K. Hohner, Peter M. Kiffney, Lorrayne Miralha, Peter Regier, Joshua Seeds, Mark River*

**Author List**

Sara Wall (0000-0002-3673-1126) U.S. Forest Service, Pacific Northwest Research Station, Olympia, WA 98512

Jana Compton (0000-0001-9833-8664) U.S. Environmental Protection Agency, Pacific Ecological Systems Division, Corvallis, OR 97333 *Corresponding author compton.jana@epa.gov

Ashley A. Coble (0000-0002-5821-5026) National Council for Air and Stream Improvement, Inc. (NCASI), Corvallis OR 97330

Beth M. Haley (0000-0003-0236-4687) U.S. Environmental Protection Agency, Pacific Ecological Systems Division, Newport, OR 97365

Jiajia Lin (0000-0002-1493-2832) Oregon Department of Environmental Quality, Water Quality Division, Portland, OR 97232

Allison Myers-Pigg (0000-0002-6905-6841) Marine and Coastal Research Laboratory, Pacific Northwest National Laboratory, Sequim, WA 98382

Justin Reale (0000-0003-3523-4782) U.S. Geological Survey, Oregon Water Science Center, Portland, OR 97204

Katie A. Wampler (0000-0002-5584-7554) Forest Ecosystems and Society, Oregon State University, Corvallis, OR 97331

Allison Swartz (0000-0002-2006-1455) Forest Ecosystems and Society, Oregon State University, Corvallis, OR 97331

Kevan Moffett (0000-0001-9564-819X) School of the Environment, Washington State University, Vancouver, WA 98686

Kevin D. Bladon (0000-0002-4182-6883) Forest Ecosystems and Society, Oregon State University, Corvallis, OR 97331

Kurt Carpenter (0000-0002-6231-8335) U.S. Geological Survey, Oregon Water Science Center, Portland, OR 97204

Heejun Chang (0000-0002-5605-6500) School of Earth, Environment, and Society, Portland State University, Portland, OR 97201

Junjie Chen (0000-0002-1396-1180), Oregon [Department of Water Resources](https://gcc02.safelinks.protection.outlook.com/?url=https%3A%2F%2Fwww.linkedin.com%2Fin%2Fchenj16up%2F&data=05%7C02%7CCompton.Jana%40epa.gov%7Cb7ab1ae6643c4154d7ae08dde3f95b96%7C88b378b367484867acf976aacbeca6a7%7C0%7C0%7C638917382300944825%7CUnknown%7CTWFpbGZsb3d8eyJFbXB0eU1hcGkiOnRydWUsIlYiOiIwLjAuMDAwMCIsIlAiOiJXaW4zMiIsIkFOIjoiTWFpbCIsIldUIjoyfQ%3D%3D%7C0%7C%7C%7C&sdata=3CtTA5XkYC6KXoBOLzo%2Bb75D8mU4ckYGLOOWm4JvC7E%3D&reserved=0), Salem, OR 97301

David Donahue (0009-0008-9826-769X) Drinking Water Protection, Eugene Water & Electric Board, Eugene, OR 97402

Chris S. Eckley (0000-0002-6986-4451) U.S. Environmental Protection Agency, Region-10, Seattle, WA 98117

Amanda K. Hohner (0000-0001-7704-4464) Department of Civil Engineering, Montana State University, Bozeman, MT 59717

Peter M. Kiffney (0000-0003-0863-0085) NOAA Fisheries, Northwest Fisheries Science Center, Fish Ecology Division, Seattle, WA 98112

Lorrayne Miralha (0000-0003-1448-9321) Department of Food, Agricultural and Biological Engineering, The Ohio State University, Columbus OH 43210

Peter Regier (0000-0002-8375-1314) Marine and Coastal Research Laboratory, Pacific Northwest National Laboratory, Sequim, WA 98382

Joshua Seeds Oregon Department of Environmental Quality, Water Quality Division, Portland, OR 97232

Mark River (0009-0005-4967-8087), Weyerhaeuser Company

Any use of trade, firm, or product names is for descriptive purposes only and does not imply endorsement by the U.S. Government.

**Supplementary Table 1**. Discrete water quality parameters represented in each broad category for Figure 3. The data are from the Water Quality Portal (WQP 2021).

| **Paper Category** | **Water Quality Parameter (CharacteristicName in WQP)** |
| --- | --- |
| Aquatic Ecology | Chlorophyll a |
|  | Chlorophyll a, uncorrected for pheophytin |
|  | Pheophytin a |
|  | Chemical oxygen demand |
|  | Biochemical oxygen demand, standard conditions |
|  | Biochemical oxygen demand, non-standard conditions |
|  | Chemical oxygen demand, (high level) |
|  | Carbonaceous biochemical oxygen demand, standard conditions |
| Metals | Metals |
|  | Cadmium |
|  | Chromium |
|  | Copper |
|  | Iron |
|  | Lead |
|  | Manganese |
|  | Molybdenum |
|  | Nickel |
|  | Zinc |
|  | Aluminum |
|  | Mercury |
| Nutrients | Orthophosphate |
|  | Total Phosphorus |
|  | Total nitrogen [nitrate + nitrite + ammonia + organic-N] |
|  | Ammonia and ammonium |
|  | Nitrite |
|  | Nitrate |
|  | Inorganic nitrogen (nitrate and nitrite) |
|  | Carbonate |
|  | Calcium |
|  | Magnesium |
|  | Sodium |
|  | Potassium |
|  | Chloride |
|  | Sulfate |
| Physical Hydrology | Stream flow |
|  | Stream flow, instantaneous |
|  | Height, gage |
|  | Depth, Secchi disk depth |

Reference

Water Quality Portal. (2021). Washington (DC): National Water Quality Monitoring Council, United States Geological Survey (USGS), Environmental Protection Agency (EPA). https://doi.org/10.5066/P9QRKUVJ, accessed August 28, 2025.
